# Timing Mechanisms for Circadian Seizures

**DOI:** 10.3390/clockssleep6040040

**Published:** 2024-10-21

**Authors:** Kristina Slabeva, Maxime O. Baud

**Affiliations:** 1 Zentrum für Experimentelle Neurologie, Inselspital, Bern University Hospital, University of Bern, 3010 Bern, Switzerland; 2 Schlaf-Wach Epilepsie Zentrum, Inselspital, Bern University Hospital, University of Bern, 3010 Bern, Switzerland

**Keywords:** circadian, sleep, epileptic seizures

## Abstract

For centuries, epileptic seizures have been noticed to recur with temporal regularity, suggesting that an underlying biological rhythm may play a crucial role in their timing. In this review, we propose to adopt the framework of chronobiology to study the circadian timing of seizures. We first review observations made on seizure timing in patients with epilepsy and animal models of the disorder. We then present the existing chronobiology paradigm to disentangle intertwined circadian and sleep–wake timing mechanisms. In the light of this framework, we review the existing evidence for specific timing mechanisms in specific epilepsy syndromes and highlight that current knowledge is far from sufficient. We propose that individual seizure chronotypes may result from an interplay between independent timing mechanisms. We conclude with a research agenda to help solve the urgency of ticking seizures.

## 1. Introduction

Most individuals with epilepsy, and their relatives, perceive seizures as occurring randomly, which leads to constant uncertainty about upcoming threats in their daily life. However, this perceived randomness belies the evidence from historical clinical observations [1,2,3] and modern chronic EEG recordings [4,5] that have unmasked cycles in seizure occurrence spanning the range of days, months and years [6]. So-called ‘seizure cycles’ can be found across different species [7] (humans, canines, rodents) as well as epilepsy localization [8] (e.g., temporal or frontal lobe) and etiologies (focal lesional or genetic), suggesting that they represent a core aspect of epilepsy. Today, seizures are seen as stochastic events whose likelihood is influenced by several factors, among which biological rhythms play a crucial role [9]. Yet, the fundamental timing mechanisms for seizures remain unknown.

Beyond the multi-scale phenomenology of seizure cycles reviewed elsewhere [7], this review focuses on the non-random circadian timing of seizures. It proposes a framework entrenched in the field of fundamental chronobiology to seek answers to a few key questions: Is the timing of seizures dependent on the environment or endogenously generated by the epileptic brain? If the latter is true, is it directly related to the clock machinery within each cell? Or is it linked to the sleep–wake cycle, which is essential to proper brain functioning? And if so, is seizure timing dependent on switches between vigilance states, or is it linked to the underlying sleep–wake homeostasis?

This review does not cover the opposite influence of epilepsy on the sleep–wake and circadian cycles, which have been reviewed elsewhere [10]. Ultimately, all aspects of multi-scale and bi-directional relationships between biological cycles and epilepsy will need to be accounted for. However, a clear research agenda can already be formulated for the study of the timing mechanisms for circadian seizure cycles.

## 2. Seizure Chronotypes

In the 19th century, Gowers described different circadian chronotypes in seizure occurrence in his patients with epilepsy: diurnal (seizures occurring during the day, presumably during wakefulness), nocturnal (seizures occurring during the night, presumably during sleep), and diffuse (no specific timing preference) [2,3]. More than a century later, a study based on chronic EEG recordings over several years revealed that a circadian modulation on seizure occurrence is indeed present in approximately 90% of patients [8]. Confirming historical observations, this circadian modulation fell into five different chronotypes, each with peak seizure likelihood at different circadian phases: three diurnal (D) chronotypes where seizures predominantly occur in the morning (D1), afternoon (D2) or evening (D3) and two nocturnal chronotypes where seizures tend to occur either during the early (N1) or late night (N2) [2,8]. These chronotypes are merely drawn from statistics on the time of occurrence but likely reflect combined chronobiological influences from the circadian and sleep-wake cycles.

Circadian patterns in seizure occurrence are not limited to humans but are also found in other species. In 1955, Franz Halberg, a founding father of chronobiology who coined the term ‘circadian’, reported circadian variations in thresholds to induce audiogenic seizures in mice [11]. In chemically induced epilepsy in nocturnal mice, spontaneously recurring seizures tended to cluster at the transition from the inactive (light) phase to the active (dark) phase, resembling a morning chronotype (D1) [12,13]. Other rodent models of epilepsy in rats [14,15,16,17,18] and mice [19,20,21] show seizure clustering during periods of inactivity and sleep, resembling nocturnal chronotypes in humans (N1-N2) with a higher risk for SUDEP [22]. Like humans, dogs with naturally occurring epilepsy may have seizures at different preferential times [7,23].

Thus, seizure timing varies across people with epilepsy and models of the disorder but tends to be conserved within individuals, constituting seizure chronotypes [8].

## 3. Timing Mechanisms for Seizures

Fundamental research into biological rhythms has historically been divided between chronobiologists focusing on the molecular mechanisms of the circadian clocks, and sleep researchers focusing on the circuits and functions of sleep, obscuring the intertwined nature of these two cycles. As a result, the lack of a holistic approach to timing mechanisms for seizures hinders the quest for biomarkers and treatments that may differ depending on the chrono-mechanism at play. Indeed, the effects of the circadian and/or the sleep–wake cycles on seizures are often conflated in the epilepsy literature, while they should be treated as related but distinct modulators of epileptic brain activity. Concretely, the term ‘circadian seizure cycles’ has been coined, irrespective of the underlying chronobiological mechanism(s). In principle, four candidate mechanisms could be involved, as they all present a ~24-h periodicity [24,25]:

(I) Environmental cyclical triggers;
(II) The circadian cycle or Process C;
(III) Sleep homeostasis or Process S, which regulates the sleep–wake cycle;
(IV) Switches in vigilance states, which occur during the sleep–wake cycle.

Their interplay is described below, as well as their potential distinct role in promoting seizures.

### 3.1. Environmental Cycles

As the earth rotates around itself in exactly 24 h, the resulting cyclical exposure to environmental triggers could influence the occurrence of seizures. For example, occasional photosensitive epilepsies and other rarer reflex epilepsies [26,27] could be associated with a non-random timing of seizure triggered during daytime. For this external influence to be the cause of a seizure cycle, the seizures need to occur almost deterministically upon trigger presentation, which is rare in epilepsy.

### 3.2. The Circadian Cycle

The circadian cycle, or rather cycles, are defined as endogenously generated biological rhythms of about 24 h, governing the timing of vital behaviours such as foraging and sleeping. Two main criteria must be met for the identification of the circadian nature of a cycle: (i) the cycle persists in a constant environment with an endogenously generated free-running period of ~24-h, and (ii) the cycle can be entrained by external factors such as light or temperature (so-called Zeitgebers) [7,28]. Circadian cycles in the brain or the body are governed by the rhythmic expression of core clock genes and are intrinsic presumably to nearly every cell [29,30,31]. They align across organs through the action of circadian hormones, which are secreted under the influence of the master clock, the suprachiasmatic nucleus [30]. In the brain, these molecular cycles modulate in the expression of synaptic receptors and signalling proteins, that influence neuronal excitability [32] and therefore conceivably also seizure likelihood.

Until the circadian nature of a cycle can be experimentally proven, the preferred term for a 24 h cycle is ‘diurnal’. Evidence from animal studies show that seizure cycles are endogenously generated [33], suggesting that the timing of seizures is governed by rhythmic biological processes on a ~24-h scale. In our opinion, it is therefore acceptable to use the terminology ‘circadian seizure cycles’, bearing in mind that the exact mechanisms are still under investigation. To be truly and solely circadian, seizures should occur at a given phase of the physiological circadian cycle regardless of external cues (i.e., be endogenous) and irrespective of momentary vigilance state (i.e., occur in sleep and wake) or sleep homeostasis (see below). Thus, some seizures may ultimately be linked to specific phases in the circadian clock machinery.

### 3.3. The Sleep–Wake Cycle

The sleep–wake cycle is apparent through switches between the asleep and awake states that are timed with a periodicity of ~24-h. When discussing the sleep–wake cycle, it is important to differentiate between the alternance in vigilance stages and the underlying sleep homeostasis—or sleep pressure—which times this alternance [25].

#### 3.3.1. Switches in Vigilance Stages

Switches in vigilance states are characterized by changes in cortical oscillations within seconds. In addition to the alternance between awake and asleep states at a ~24 h period, an ultradian (<24 h period) cycle is nested in the asleep period, consisting in the alternance between sleep stages, namely non-rapid-eye-movement (NREM) and rapid-eye-movement (REM) sleep. NREM sleep is characterized by sleep spindles and slow waves, while REM sleep is characterized by low voltage, faster and desynchronized wake-like EEG patterns [34]. Since vigilance states are non-uniformly distributed over the circadian cycle, they too could explain preferential seizure timing [35]. It is conceivable that specific cortical oscillations may increase or decrease seizure likelihood [36]. For example, increased synchronisation across the thalamocortical network during NREM sleep may promote seizures [37]. Thus, some seizures may be intimately linked to sleep stages and specific brain oscillations.

#### 3.3.2. Sleep Homeostasis

Sleep homeostasis refers to the process that regulates the balance between sleep and wakefulness [24]. A sleep ‘homeostat’ keeps track of the cumulative time awake and proportionally imposes increasing sleep pressure reflecting the need to sleep. While the changes in brain circuits dynamics underlying brain state transitions are well understood, the sleep homeostat remains a quest of fundamental neurobiology [25]. Putatively, neurons exhaust some reserve during wake that must be replenished during sleep. Synapses seem to undergo systematic changes with time spent awake [32,38,39]. Regardless of the fundamental mechanisms of sleep homeostasis, seizures could theoretically result from build-up in sleep pressure, in which case they should mostly occur after prolonged wakefulness, possibly independently of a circadian phase or the momentary brain state. Thus, some seizures may ultimately be linked to synaptic changes that occur with time spent awake. Alternatively, high sleep pressure may favour the emergence of drowsiness and its associated cortical oscillations, which could precipitate seizures.

### 3.4. The Two-Process Model of Sleep (and Seizures)

**Figure 1:**
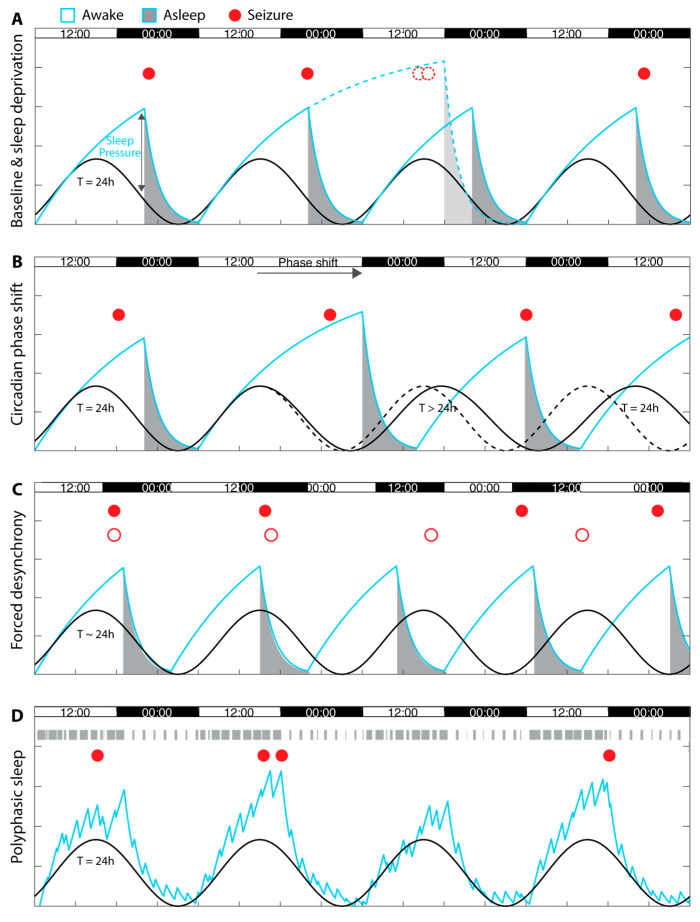
**Two-process model of seizure timing.** (**A**) Hypothetical patient with seizures linked to increases in sleep homeostasis (Process S, cyan curve), who has two additional seizures (dotted empty dots) after a 32 h sleep deprivation (dotted cyan line). (**B**) Hypothetical patient with seizures during wakefulness or during sleep linked to increases in sleep homeostasis. A circadian phase shift is followed by an entrainment of the sleep–wake cycle on a different schedule, and seizures as well as sleep are decoupled from Process C (black curve) for a few days. (**C**) Two hypothetical subjects undergoing forced desynchrony on a 10-h light/10-h dark schedule. In the subject with seizures linked to Process S (full dots), seizures occur with peaks in sleep pressure at the end of the scheduled active period (light). In the subject with seizures linked to Process C (empty dots), seizures occur at the circadian peaks, independently of the light-dark schedule. (**D**) Subject with polyphasic sleep (e.g., mouse model, sleep bout as grey boxes on top), in which Process S shows more variations at different circadian phases. As a result, seizures linked to Process S may occur at more than one circadian phase.

The ‘Two process model of sleep’ [24,25] describes sleep timing as being dependent on the two tightly coupled oscillators mentioned above: the circadian cycle (Process C) which promotes wakefulness at times when it is needed to exploit the environment and sleep homeostasis (Process S) which promotes sleep as a restorative process (Figure 1). Under most circumstances these processes are tightly coupled and jointly time sleep, and perhaps seizures. In the model, Process S is described as a relaxation oscillator entrained by a harmonic oscillator (Process C). Unlike Process C, Process S can be engaged well-beyond 24 h, in case of necessary or voluntary sleep deprivation (Figure 1A). Indeed, the ability to post-pone sleep beyond its usual timing has undoubtful ecological value, while the rigidity of Process C helps maintain alignment with the external 24-h light–dark cycle. Currently, it is unknown whether Process S or C have a greater influence on seizure timing, but chronobiological manipulations may help disentangle their contribution.

## 4. Experimental Paradigms

Association studies are insufficient in disentangling which of these mechanisms may be causally linked to the circadian timing of seizures because the candidate mechanisms co-vary on the same timescale (~24 h). Several experimental paradigms established in the field of chronobiology may help gain clarity by either manipulating one of the two processes or their interplay. Some can be used in people with epilepsy, while others only apply to animal models of the disorder.

### 4.1. Constant Environment

A key chronobiological manipulation that can confirm the endogenous nature of a seizure cycle is to place the subject in a constant environment [28]. If the seizure cycle persists, it means that it is endogenously generated and does not depend solely on the cyclical presentation of external triggers. This paradigm helps rule out a role of the environment but does not necessarily help attribute a specific role to the circadian or sleep–wake cycle. Indeed, in a constant environment, the physiological circadian cycles persist throughout the body with a periodicity of ~24 h—the ‘free-running’ circadian period—entraining, among others, the sleep–wake cycle.

### 4.2. Sleep Manipulations

Given the flexibility of the sleep–wake cycle, sleep or wake can be imposed at unusual circadian phases for experimental purposes. In humans, wakefulness or naps can be imposed on a voluntary basis, while in animals, sleep deprivation manipulations can be applied.

#### 4.2.1. Sleep Deprivation

Prolonged sleep deprivation of >24 h in humans and >6 h in rodents has been associated with stress [40], but shorter manipulations likely fall within physiological range. Imposing wakefulness when sleep is expected may help show that seizures do not depend on an underlying vigilance state. Prolonging wake can also increase sleep pressure and may lead to increases in seizures (Figure 1A). In fact, sleep deprivation is commonly used in clinical practice to trigger seizures, although the evidence to support this practice is currently lacking [41].

#### 4.2.2. Nap

A second use of sleep deprivation is to promote recovery sleep at times when sleep is not expected. In clinical practice again, patients are often instructed to sleep less on the night before they present to the hospital for a routine EEG, such that the likelihood of falling asleep during the exam is higher. If seizures are triggered by sleep even during naps, sleep-bound seizures can be diagnosed. In many other cases, though, sleep ‘activates’ interictal epileptiform discharges without necessarily provoking seizures [41,42,43].

### 4.3. Circadian Manipulations

Dissociating the timing of sleep from the circadian cycle with sleep deprivation or naps can be seen as a punctual desynchrony of the two cycles. Another mean of forcing punctual desynchrony between the two cycles is to intervene on the circadian cycle and let the sleep–wake cycle follow.

#### 4.3.1. Phase Shift

By changing the light–dark schedule, for example when traveling across longitudes, humans impose on themselves a phase-shift in the circadian cycle; that is, the endogenous circadian cycle is no longer aligned with the external Zeitgeber (i.e., light). As a rule of thumb, it takes one day to recover from each hour of jetlag imposed. Such manipulations can also be used experimentally to determine whether seizures remain in phase with the lagging circadian cycle [44] or follow the sleep–wake cycle (Figure 1B).

#### 4.3.2. Dampened Amplitude

By imposing a constant Zeitgeber without a cycle (e.g., constant light environment) the circadian cycle will tend to lengthen and weaken. Such manipulation can help reveal the importance of the strength of the circadian cycles.

#### 4.3.3. Clockless Animals

Unlike the sleep–wake cycle, the circadian cycle can be switched off in experimental animals by mutations in core clock genes [31]. This allows researchers to test the role of the sleep–wake cycle independently from the circadian cycle [45].

### 4.4. Desynchrony

For a complete desynchrony to occur between co-existing sleep–wake and circadian cycles, longer chronobiological manipulations can be used to set the sleep–wake cycle on one schedule, while the circadian cycle is free-running [46]. *Spontaneous* desynchrony is observed when humans are kept long enough (e.g., >8 weeks) in a constant environment in which they can time their rest. In such circumstances, the self-selected sleep–wake times (Process S) may uncouple from the free-running circadian cycles (Process C; e.g., core body temperature) [46]. Experimental protocols of *forced* synchrony (humans and animals) and constant routine (humans) were developed to attain such uncoupling more rapidly.

#### 4.4.1. Forced Desynchrony

By scheduling individuals to rest and be active on a non-circadian period—either too short (e.g., ≤20 h) or too long (e.g., ≥28 h)—sleep and wakefulness are progressively distributed across the entire rather than a portion of the circadian cycle [46]. Indeed, the sleep–wake cycle is entrained by the light–dark schedule on a periodicity outside the entrainment range of the circadian cycle [46]. In the so-called T20 paradigm in humans [46], sleep mostly occurs over a 10-h night, and wake over a 10-h day. In such a non-circadian environment, the circadian cycle maintains its free-running ~24 h period (Figure 1C). In the T20 paradigm in rodents, though, the polyphasic sleep–wake cycle will often exhibit both a 20-h period imposed by the environment, *and* a 24-h period entrained by the circadian cycle [47]. Thus, in rodents under forced desynchrony the sleep–wake and circadian cycles co-exist but have different (mixed) periods, leading to an interferential pattern in locomotor activity [47]. Specifically, animals are least or most active when certain phases of Process C and S align or misalign for a few hours. Such manipulations may help assign seizures to one or the other periodicity.

#### 4.4.2. Constant Routine

In humans, unnatural polyphasic sleep with sleep bouts of only minutes to a few hours is imposed in some professions and can be used experimentally in so-called ‘constant routine’ experiments [48], but it is rarely used in epilepsy research [49]. Although constant routines cannot be imposed onto rodents, they naturally present polyphasic sleep, which can further help distinguish between the influence of the sleep–wake and circadian cycles in timing seizures, as each vigilance state is naturally occurring at nearly all circadian phases (Figure 1D).

## 5. Current Evidence for Timing Mechanisms for Seizures

Although research typically focuses either on the circadian or the sleep–wake cycle, many separate pieces of evidence for one or the other mechanism in specific seizure chronotypes have been found, both in clinical epileptology and epilepsy research, which we here discuss together.

### 5.1. Evidence for the Endogenous Timing of Seizures

Rodent models of epilepsy helped uncover the endogenous nature of circadian seizure cycles. Quigg et al. demonstrated in a rat model of temporal-lobe epilepsy that the preferred timing of seizure occurrence persisted in animals placed in a constant dark environment [33], indicating that the timing of seizure occurrences in these animals is endogenously generated. This simple experiment (switching the light off!) essentially rules out the sole implication of environmental cyclical factors for seizure timing. Despite its significance in fulfilling the first criterion for a truly circadian seizure cycle, this finding has not been replicated in the past 25 years.

### 5.2. Evidence for the Role of the Circadian Cycle in Seizure Timing

Later studies imposed a phase-shift on animals with epilepsy, showing that seizure cycles can be entrained by Zeitgebers, fulfilling the second criterion for a truly circadian seizure cycle [44]. Additionally, the use of transgenic models with knock outs for different core clock genes showed that manipulation of the core clock machinery can cause epilepsy and might be linked to fluctuations in seizure likelihood. For example, ablation of *clock* in pyramidal cells resulted in spontaneous sleep-bound seizures [21], while ablation of *clock* in inhibitory interneurons increased seizure thresholds [50]. Ablation of *bmal-1* [51] and its downstream targets [52,53] abolished the circadian variation in seizure threshold for electrically and chemically induced seizures. Surprisingly, none of these studies characterized seizure timing.

### 5.3. Evidence for the Role of Sleep Homeostasis in Seizure Timing

Despite its routine clinical use, a definitive causal link between sleep deprivation and increased seizure occurrence is still lacking, as highlighted by a recent review [41].

#### 5.3.1. Sleep Homeostasis and Cortical Excitability

Limited evidence in humans suggests that cortical excitability assessed via transcranial magnetic evoked potentials fluctuates with the circadian cycle but also increases with time spent awake [54,55]. In animals, a body of electrophysiological measurements support the idea that cortical excitability varies with time spent awake [56]. If variations in cortical excitability directly influence seizure likelihood, then the homeostatic variation in cortical characteristics represents a credible mechanism contributing to seizure timing.

#### 5.3.2. Clinical Evidence

Studies in epilepsy patients have reported conflicting results with some studies suggesting a positive correlation between seizure occurrence and sleep pressure [43,57,58], while others report no such correlation [59,60,61,62]. Notably, these observational studies have several limitations, including a lack of control for confounding factors known to influence seizure occurrence in humans (e.g., stress or alcohol consumption) and, more crucially, a failure to control for the preceding amount of sleep or naps in these patients [41].

#### 5.3.3. Experimental Evidence

Several studies have examined the relationship between sleep pressure and epilepsy in animal models under controlled laboratory conditions. In the 1970s, several REM-sleep deprivation experiments in non-epileptic mice, rats and cats consistently showed a decrease in seizure thresholds tested by electroshocks [63,64,65,66]. In the 1980s, these findings were replicated for REM- and/or NREM-sleep deprivation and extended to the fact that reduced thresholds to penicillin or electrical stimulation were found in all vigilance stages, except for REM sleep [67,68]. These early works on changes in excitability and seizure thresholds were followed by similar experiments in models of chronic epilepsy.

In a rat model of absence epilepsy, sleep deprivation led to increased seizure rates [69]. In a genetic mouse model of severe fatal epilepsy with insomnia [70], daily sleep deprivation for 4 h over 5 days increased the likelihood of status epilepticus and SUDEP [71]. Cuddapah et al. [72] have associated sleep loss with increased seizure rate in drosophila. They specifically showed that bidirectional optogenetic manipulation of sleep promoting networks can improve or worsen seizure rates.

In summary, while human studies have provided insufficient evidence for a causal link between sleep homeostasis and seizures [41], animal models have consistently showed that sleep deprivation is associated with increased susceptibility to various epilepsy-related outcomes, including seizure thresholds [64,68], seizure rates [69,71] and the occurrence of SUDEP [71]. None of these studies characterized the potential impact of longitudinal fluctuations in sleep pressure on seizure timing though, for example by modelling process S. 

### 5.4. Evidence for the Role of Vigilance Stages in Seizure Timing

Specific epilepsy syndromes like sleep-related hypermotor epilepsy [73] (with seizure occurring during sleep), juvenile myoclonic epilepsy and grand mal seizures upon awakening (with seizures often occurring within 2 h after awakening) [74], or other genetic epilepsies [75], suggest a connection between epilepsy and specific vigilance stages. Ng et al. showed evidence in a meta-analysis of 42 studies with a total of 1458 patients that focal and generalized seizures are most commonly observed during NREM sleep and very rarely during REM sleep [35]. Complementary results have been found in animal models of epilepsy where the seizure threshold was higher during REM sleep compared to NREM sleep [65], and enhancing the characteristic oscillations of REM sleep showed a protective effect towards seizures [76,77,78].

## 6. The Temporal Ictal–Interictal Relationship

So far, we have only reported results from the study of seizure timing, which is the obvious manifestation of epilepsy. However, many of these studies and others have also investigated the timing of interictal epileptiform discharges (IEDs). Conceptually, IEDs may be under the same or a different influence from the presented chrono-mechanisms. Evidence suggests, though, that sleep plays a particularly important role in increasing the rates of IEDs. IEDs are invariably most frequent in NREM sleep, while REM sleep seems to have a protective effect [79,80,81,82]. More specifically, IEDs are more prone to occur during cortical slow wave (0.5–4 Hz) oscillations observable during the N2 and N3 stages of NREM sleep [81,83,84,85,86,87], putatively due to increased neuronal synchrony during these vigilance states [84,87]. Interestingly, the permissive effect of NREM sleep on IEDs can also be found in patients with diurnal seizure chronotypes [8] and was shown experimentally to be independent of the circadian cycle [49].

## 7. Summary and Next Steps

Taken together, clinical and experimental observations suggest that rates of IEDs are mostly modulated by the sleep–wake cycle, whereas different seizure chronotypes may be under variable co-modulation by both the circadian and the sleep–wake cycles.

### 7.1. Weaknesses in Current Research

While statistical observations on the distributions of seizures in association to vigilance stages or circadian time are widespread, experimental work aiming at manipulating these distributions is currently lacking. Much of the experimental work in epilepsy has focused on ictogenesis; that is, the immediate network or cellular dynamics that may precipitate seizures. Crucially, few of the studies listed here actually studied seizure timing. Rather, they have focused on seizure thresholds, most at a single timepoint and some [51] at multiple timepoints, using variable means to induce acute seizures, such as kainic acid [52], pilocarpine [50], pentylenetetrazole or electroshocks [51] (Table 1). Alternatively, some have focused on seizure severity [50,52] or progression [50,52] with repeated application of a chemo-convulsant. Chemo-convulsive seizures are variable in wild-type animals and do not necessarily directly relate to time-varying seizure likelihood, a more frequent clinical phenomenon. To our knowledge, of the few studies [33,44] that have highlighted and studied non-random seizure timing, none have thought to disentangle the underlying mechanisms by leveraging the arsenal of available chronobiological manipulations presented here. This represents a major knowledge gap that must be addressed to prevent ticking seizures.

### 7.2. Research Agenda

The importance of seizure chronotypes in people with epilepsy has been recognized for more than a century [1,2,3] and recently characterized quantitatively [4,5,6,7,8]. Towards a mechanistic understanding of this phenomenon, significant advances will require animal experimentation. 

Acknowledgement of the fundamental advances in the field of chronobiology is the first step towards clear concepts in pursuing the chronobiological mechanisms in epilepsy. We believe this review may help build bridges between these fields. For true experimentation with seizure timing, an armamentarium of experimental paradigms from fundamental chronobiology and sleep research are available and are listed above. By design, modern experimentation with seizure timing requires long to very long experiments (months). The core question at hand is not whether epilepsy is present or not or whether seizure duration or severity may vary, but whether the temporal distribution of dozens to hundreds of seizures, analysed within and across several individuals and experimental paradigms, may be modulated by one or more timing mechanisms. In addition to the challenge of conducting months-long chronobiological experiments, the large amounts of data generated invite machine-learning techniques to supplement classical statistics and help with the large analyses required. Within this established framework, the study of different animal models with different seizure timing will help rapidly advance the understanding of distinct seizure chronotypes.

## Figures and Tables

**Table 1:** Some published chronobiological experiments on seizure timing mechanisms. This list does not include purely observational studies of seizure timing or the effect of seizures on sleep or the circadian cycle. S: manipulation on sleep states or Process S, C: manipulation on the circadian cycle (process C), and FD: manipulation to force desynchrony between Process S and C. CE: constant environment, PS: phase-shift, and KO: knock out of one of the clock-genes. Sz: seizures, IED: interictal epileptiform discharges, and Thr: seizure threshold. PTZ: pentylenetetrazole, KA: kainic acid, PIL: pilocarpine, ES: electroshock and AD: afterdischarges elicited by kindling electrical stimulation. RSD: REM-sleep sleep deprivation, TSD: total sleep deprivation, and GSD: gentle sleep deprivation. ✓: experiment or measurement done. (✓): measurement partially done. ☓: Experiment or measurement not done.

First Author	Year	Model or Syndrome	Seizure Chronotype	Manipulation			Read Out			Comment
				S	C	FD				
							Timing		Thr	
							Sz	IED		
Quigg [33]	2000	TLE in rat	Resting phase	☓	✓ CE	☓	✓	☓	☓	First demonstration of the endogenous nature of seizure timing.
Debski [44]	2020	Pilocarpine TLE in mice	Resting phase	☓	✓ PS	☓	✓	☓	✓ PTZ	Mild diurnal fluctuation of the PTZ threshold. Seizure diurnal distribution follows an 8 h shift in the light–dark schedule.
Li [21]	2017	*Clock* KO mice	Sleep-bound	☓	✓ KO	☓	(✓)	☓	✓ PTZ	Clock gene KO in pyramidal neurons can lead to sleep-bound seizures and decreased seizure threshold, but not when the KO is in PV interneurons.
Deng [50]	2024	*Clock* KO mice	Acute szs	☓	✓ KO	☓	☓	☓	✓ PIL	Clock gene KO in interneurons increases latency to pilocarpine-induced seizures.
Gerstner [51]	2014	*Bmal-1* KO mice	Acute szs	☓	✓ KO	☓	☓	☓	✓ ES	Loss of the circadian modulation of the electroshock seizure Thr.
Zhang [52]	2021	*Rev-erbα* KO mice	Not specified	☓	✓ KO	☓	☓	☓	✓ KA	Loss of the circadian modulation of the severity of KA-induced seizures.
Gachon [53]	2004	*DBP/HLF/TEF* KO mice	Early sleep	☓	✓ KO	☓	☓	☓	☓	Serendipitous finding of audiogenic seizures occurring during early sleep.
Cohen [64,65,66] Owen [63]	1965 1970 1970	Non-epileptic rats Non-epileptic cats/ice Non-epileptic rats		✓ RSD	☓	☓	☓	☓	✓ ES	Decreased threshold after REM-sleep deprivation.
Shouse [67,68]	1982 1988	Non-epileptic rats Non-epileptic cats		✓ 24–72 h TSD	☓	☓	☓	☓	✓ AD PEN	Decreased threshold after sleep deprivation upon penicilline or electrical stimulation in all vigilance stages.
Grahnstedt [88]	1986	Non-epileptic rats		✓ 96 h TSD	☓	☓	☓	☓	✓ AD	Decreased threshold after sleep deprivation
Drinkenburg [69]	1995	Absence epilepsy in rats	Not specified	✓ 12 h TSD	☓	☓	☓	✓	☓	Increase of IED rates at the onset of total sleep deprivation.
Konduru [71]	2021	*Kv1.1* KO mice	Not specified	✓ 4 h GSD	☓	☓	☓	☓	☓	Dramatic increase in seizure and SUDEP rates during 5 days of 4-h SD.

## References

[1] Griffiths G.M., Fox J.T. (1938). Rhythm in Epilepsy. Lancet.

[2] Langdon-Down M., Brain W.R. (1929). Time of Day in Relation to Convulsions in Epilepsy. Lancet.

[3] Gowers W.R. (1881). Epilepsy and Other Chronic Convulsive Diseases: Their Causes, Symptoms and Treatment.

[4] Baud M.O., Kleen J.K., Mirro E.A., Andrechak J.C., King-Stephens D., Chang E.F., Rao V.R. (2018). Multi-day rhythms modulate seizure risk in epilepsy. Nat. Commun..

[5] Karoly P.J., Freestone D.R., Boston R., Grayden D.B., Himes D., Leyde K., Seneviratne U., Berkovic S., O’brien T., Cook M.J. (2016). Interictal spikes and epileptic seizures: Their relationship and underlying rhythmicity. Brain.

[6] Leguia M.G., Rao V.R., Kleen J.K., Baud M.O. (2021). Measuring synchrony in bio-medical timeseries. Chaos Interdiscip. J. Nonlinear Sci..

[7] Karoly P.J., Rao V.R., Gregg N.M., Worrell G.A., Bernard C., Cook M.J., Baud M.O. (2021). Cycles in epilepsy. Nat. Rev. Neurol..

[8] Leguia M.G., Andrzejak R.G., Rummel C., Fan J.M., Mirro E.A., Tcheng T.K., Rao V.R., Baud M.O. (2021). Seizure Cycles in Focal Epilepsy. JAMA Neurol..

[9] Baud M.O., Proix T., Rao V.R., Schindler K. (2020). Chance and risk in epilepsy. Curr. Opin. Neurol..

[10] Khan S., Nobili L., Khatami R., Loddenkemper T., Cajochen C., Dijk D.-J., Eriksson S.H. (2018). Circadian rhythm and epilepsy. Lancet Neurol..

[11] Halberg F., Bittner J.J., Gully R.J., Albrecht P.G., Brackney E.L. (1955). 24-Hour Periodicity and Audiogenic Convulsions in I Mice of Various Ages. Exp. Biol. Med..

[12] Pitsch J., Becker A.J., Schoch S., Müller J.A., de Curtis M., Gnatkovsky V. (2017). Circadian clustering of spontaneous epileptic seizures emerges after pilocarpine-induced status epilepticus. Epilepsia.

[13] Lisgaras C.P., Scharfman H.E. (2022). Robust chronic convulsive seizures, high frequency oscillations, and human seizure onset patterns in an intrahippocampal kainic acid model in mice. Neurobiol. Dis..

[14] Matzen J., Buchheim K., Holtkamp M. (2012). Circadian dentate gyrus excitability in a rat model of temporal lobe epilepsy. Exp. Neurol..

[15] Tchekalarova J., Pechlivanova D., Itzev D., Lazarov N., Markova P., Stoynev A. (2010). Diurnal rhythms of spontaneous recurrent seizures and behavioral alterations of Wistar and spontaneously hypertensive rats in the kainate model of epilepsy. Epilepsy Behav..

[16] Hellier J.L., Dudek F.E. (1999). Spontaneous motor seizures of rats with kainate-induced epilepsy: Effect of time of day and activity state. Epilepsy Res..

[17] Baud M.O., Ghestem A., Benoliel J.J., Becker C., Bernard C. (2019). Endogenous multidien rhythm of epilepsy in rats. Exp. Neurol..

[18] Quigg M., Straume M., Menaker M., Bertram E.H. (1998). Temporal distribution of partial seizures: Comparison of an animal model with human partial epilepsy. Ann. Neurol..

[19] Fenoglio-Simeone K.A., Wilke J.C., Milligan H.L., Allen C.N., Rho J.M., Maganti R.K. (2009). Ketogenic diet treatment abolishes seizure periodicity and improves diurnal rhythmicity in epileptic Kcna1-null mice. Epilepsia.

[20] Wright S., Wallace E., Hwang Y., Maganti R. (2016). Seizure phenotypes, periodicity, and sleep–wake pattern of seizures in Kcna-1 null mice. Epilepsy Behav..

[21] Li P., Fu X., Smith N.A., Ziobro J., Curiel J., Tenga M.J., Martin B., Freedman S., Rio C.A.C.-D., Oboti L. (2017). Loss of CLOCK Results in Dysfunction of Brain Circuits Underlying Focal Epilepsy. Neuron.

[22] Purnell B.S., Petrucci A.N., Li R., Buchanan G.F. (2021). The effect of time-of-day and circadian phase on vulnerability to seizure-induced death in two mouse models. J. Physiol..

[23] Gregg N.M., Nasseri M., Kremen V., Patterson E.E., Sturges B.K., Denison T.J., Brinkmann B.H., Worrell G.A. (2020). Circadian and multiday seizure periodicities, and seizure clusters in canine epilepsy. Brain Commun..

[24] Borbely A.A. (1982). A Two Process Model of Sleep Regulation. Hum. Neurobiol..

[25] Borbély A.A., Daan S., Wirz-Justice A., Deboer T. (2016). The two-process model of sleep regulation: A reappraisal. J. Sleep Res..

[26] Koepp M.J., Caciagli L., Pressler R.M., Lehnertz K., Beniczky S. (2016). Reflex seizures, traits, and epilepsies: From physiology to pathology. Lancet Neurol..

[27] Strzelecka J., Mazurkiewicz D.W., Skadorwa T., Gąsior J.S., Jóźwiak S. (2022). Photo-Dependent Reflex Seizures—A Scoping Review with Proposal of Classification. J. Clin. Med..

[28] Fuller P.M., Lu J., Saper C.B. (2009). Standards of evidence in chronobiology: A response. J. Circadian Rhythm..

[29] Mure L.S., Le H.D., Benegiamo G., Chang M.W., Rios L., Jillani N., Ngotho M., Kariuki T., Dkhissi-Benyahya O., Cooper H.M. (2018). Diurnal transcriptome atlas of a primate across major neural and peripheral tissues. Science.

[30] Saper C.B., Scammell T.E., Lu J. (2005). Hypothalamic regulation of sleep and circadian rhythms. Nature.

[31] Mohawk J.A., Green C.B., Takahashi J.S. (2012). Central and Peripheral Circadian Clocks in Mammals. Neuroscience.

[32] Noya S.B., Colameo D., Brüning F., Spinnler A., Mircsof D., Opitz L., Mann M., Tyagarajan S.K., Robles M.S., Brown S.A. (2019). The forebrain synaptic transcriptome is organized by clocks but its proteome is driven by sleep. Science.

[33] Quigg M., Clayburn H., Straume M., Menaker M., Bertram E.H. (2000). Effects of circadian regulation and rest-activity state on spontaneous seizures in a rat model of limbic epilepsy. Epilepsia.

[34] Adamantidis A.R., Herrera C.G., Gent T.C. (2019). Oscillating circuitries in the sleeping brain. Nat. Rev. Neurosci..

[35] Ng M., Pavlova M. (2013). Why Are Seizures Rare in Rapid Eye Movement Sleep? Review of the Frequency of Seizures in Different Sleep Stages. Epilepsy Res. Treat..

[36] Frauscher B., Gotman J. (2019). Sleep, oscillations, interictal discharges, and seizures in human focal epilepsy. Neurobiol. Dis..

[37] Steriade M. (2005). Sleep, epilepsy and thalamic reticular inhibitory neurons. Trends Neurosci..

[38] Tononi G., Cirelli C. (2006). Sleep function and synaptic homeostasis. Sleep Med. Rev..

[39] Brüning F., Noya S.B., Bange T., Koutsouli S., Rudolph J.D., Tyagarajan S.K., Cox J., Mann M., Brown S.A., Robles M.S. (2019). Sleep-wake cycles drive daily dynamics of synaptic phosphorylation. Science.

[40] Mongrain V., Hernandez S.A., Pradervand S., Dorsaz S., Curie T., Hagiwara G., Gip P., Heller H.C., Franken P. (2010). Separating the Contribution of Glucocorticoids and Wakefulness to the Molecular and Electrophysiological Correlates of Sleep Homeostasis. Sleep.

[41] Rossi K.C., Joe J., Makhija M., Goldenholz D.M. (2020). Insufficient Sleep, Electroencephalogram Activation, and Seizure Risk: Re-Evaluating the Evidence. Ann. Neurol..

[42] Carpay J.A., de Weerd A.W., Schimsheimer R.J., Stroink H., Brouwer O.F., Peters A.C.B., van Donselaar C.A., Geerts A.T., Arts W.F.M. (1997). The Diagnostic Yield of a Second EEG After Partial Sleep Deprivation: A Prospective Study in Children with Newly Diagnosed Seizures. Epilepsia.

[43] Samsonsen C., Sand T., Bråthen G., Helde G., Brodtkorb E. (2016). The impact of sleep loss on the facilitation of seizures: A prospective case-crossover study. Epilepsy Res..

[44] Debski K.J., Ceglia N., Ghestem A., Ivanov A.I., Brancati G.E., Bröer S., Bot A.M., Müller J.A., Schoch S., Becker A. (2020). The circadian dynamics of the hippocampal transcriptome and proteome is altered in experimental temporal lobe epilepsy. Sci. Adv..

[45] Franken P., Dijk D.-J. (2009). Circadian clock genes and sleep homeostasis. Eur. J. Neurosci..

[46] Wang W., Yuan R.K., Mitchell J.F., Zitting K.-M., Hilaire M.A.S., Wyatt J.K., Scheer F.A.J.L., Wright K.P., Brown E.N., Ronda J.M. (2023). Desynchronizing the sleep-wake cycle from circadian timing to assess their separate contributions to physiology and behaviour and to estimate intrinsic circadian period. Nat. Protoc..

[47] Hasan S., Foster R.G., Vyazovskiy V.V., Peirson S.N. (2018). Effects of circadian misalignment on sleep in mice. Sci. Rep..

[48] Duffy J.F., Dijk D.-J. (2002). Getting Through to Circadian Oscillators: Why Use Constant Routines?. J. Biol. Rhythm..

[49] Pavlova M.K., Shea S.A., Scheer F.A.J.L., Bromfield E.B. (2009). Is there a circadian variation of epileptiform abnormalities in idiopathic generalized epilepsy?. Epilepsy Behav..

[50] Deng L., Jiang H., Lin J., Xu D., Qi A., Guo Q., Li P.-P., Wang X., Liu J.S., Fu X. (2024). Clock knockout in inhibitory neurons reduces predisposition to epilepsy and influences anxiety-like behaviors in mice. Neurobiol. Dis..

[51] Gerstner J.R., Smith G.G., Lenz O., Perron I.J., Buono R.J., Ferraro T.N. (2014). BMAL1 controls the diurnal rhythm and set point for electrical seizure threshold in mice. Front. Syst. Neurosci..

[52] Zhang T., Yu F., Xu H., Chen M., Chen X., Guo L., Zhou C., Xu Y., Wang F., Yu J. (2021). Dysregulation of REV-ERBα impairs GABAergic function and promotes epileptic seizures in preclinical models. Nat. Commun..

[53] Gachon F., Fonjallaz P., Damiola F., Gos P., Kodama T., Zakany J., Duboule D., Petit B., Tafti M., Schibler U. (2004). The loss of circadian PAR bZip transcription factors results in epilepsy. Genes Dev..

[54] Huber R., Mäki H., Rosanova M., Casarotto S., Canali P., Casali A.G., Tononi G., Massimini M. (2013). Human Cortical Excitability Increases with Time Awake. Cereb. Cortex.

[55] Ly J.Q.M., Gaggioni G., Chellappa S.L., Papachilleos S., Brzozowski A., Borsu C., Rosanova M., Sarasso S., Middleton B., Luxen A. (2016). Circadian regulation of human cortical excitability. Nat. Commun..

[56] Vyazovskiy V.V., Cirelli C., Pfister-Genskow M., Faraguna U., Tononi G. (2008). Molecular and electrophysiological evidence for net synaptic potentiation in wake and depression in sleep. Nat. Neurosci..

[57] Haut S.R., Hall C.B., Masur J., Lipton R.B. (2007). Seizure occurrence: Precipitants and prediction. Neurology.

[58] Rajna P., Veres J. (1993). Correlations Between Night Sleep Duration and Seizure Frequency in Temporal Lobe Epilepsy. Epilepsia.

[59] Dell K.L., Payne D.E., Kremen V., Maturana M.I., Gerla V., Nejedly P., Worrell G.A., Lenka L., Mivalt F., Boston R.C. (2021). Seizure likelihood varies with day-to-day variations in sleep duration in patients with refractory focal epilepsy: A longitudinal electroencephalography investigation. EClinicalMedicine.

[60] Cobabe M.M., Sessler D.I., Nowacki A.S., O’Rourke C., Andrews N., Foldvary-Schaefer N. (2015). Impact of sleep duration on seizure frequency in adults with epilepsy: A sleep diary study. Epilepsy Behav..

[61] Malow B.A., Passaro E., Milling C., Minecan D.N., Levy K. (2002). Sleep deprivation does not affect seizure frequency during inpatient video-EEG monitoring. Neurology.

[62] Stirling R.E., Hidajat C.M., Grayden D.B., D’souza W.J., Naim-Feil J., Dell K.L., Schneider L.D., Nurse E., Freestone D., Cook M.J. (2022). Sleep and seizure risk in epilepsy: Bed and wake times are more important than sleep duration. Brain.

[63] Owen M., Bliss E. (1970). Sleep loss and cerebral excitability. Am. J. Physiol.-Leg. Content.

[64] Cohen H.B., Dement W.C. (1965). Sleep: Changes in Threshold to Electroconvulsive Shock in Rats after Deprivation of “Paradoxical” Phase. Science.

[65] Cohen H., Thomas J., Dement W.C. (1970). Sleep stages, REM deprivation and electroconvulsive threshold in the cat. Brain Res..

[66] Cohen H.B., Dement W.C. (1970). Prolonged tonic convulsions in REM deprived mice. Brain Res..

[67] Shouse M.N. (1988). Sleep deprivation increases thalamocortical excitability in the somatomotor pathway, especially during seizure-prone sleep or awakening states in feline seizure models. Exp. Neurol..

[68] Shouse M.N., Sterman M.B. (1982). Acute sleep deprivation reduces amygdala-kindled seizure thresholds in cats. Exp. Neurol..

[69] Drinkenburg W.H.I.M., Coenen A.M.L., Vossen J.M.H., Luijtelaar E.L.J.M. (1995). van. Sleep Deprivation and Spike-Wave Discharges in Epileptic Rats. Sleep.

[70] Iyer S.H., Matthews S.A., Simeone T.A., Maganti R., Simeone K.A. (2018). Accumulation of rest deficiency precedes sudden death of epileptic Kv1.1 knockout mice, a model of sudden unexpected death in epilepsy. Epilepsia.

[71] Konduru S.S., Pan Y., Wallace E., Pfammatter J.A., Jones M.V., Maganti R.K. (2021). Sleep Deprivation Exacerbates Seizures and Diminishes GABAergic Tonic Inhibition. Ann. Neurol..

[72] Cuddapah P.A., Hsu C.T., Li P., Shah H.M., Saul C., Killiany S., Shon J., Yue Z., Gionet G., Putt M.E. (2023). Sleepiness, not total sleep amount, increases seizure risk. bioRxiv.

[73] Tinuper P., Bisulli F., Cross J., Hesdorffer D., Kahane P., Nobili L., Provini F., Scheffer I.E., Tassi L., Vignatelli L. (2016). Definition and diagnostic criteria of sleep-related hypermotor epilepsy. Neurology.

[74] Xu L., Guo D., Liu Y.-Y., Qiao D.-D., Ye J.-Y., Xue R. (2018). Juvenile myoclonic epilepsy and sleep. Epilepsy Behav..

[75] Winawer M.R., Shih J., Beck E.S., Hunter J.E., Epstein M.P. (2016). Genetic effects on sleep/wake variation of seizures. Epilepsia.

[76] Shouse M.N., Siegel J.M., Wu M.F., Szymusiak R., Morrison A.R. (1989). Mechanisms of seizure suppression during rapid-eye-movement (REM) sleep in cats. Brain Res..

[77] Kumar P., Raju T.R. (2001). Seizure susceptibility decreases with enhancement of rapid eye movement sleep. Brain Res..

[78] Miller J.W., Turner G.M., Gray B.C. (1994). Anticonvulsant effects of the experimental induction of hippocampal theta activity. Epilepsy Res..

[79] Giacomini T., Luria G., D’amario V., Croci C., Cataldi M., Piai M., Nobile G., Bruni O., Consales A., Mancardi M.M. (2022). On the role of REM sleep microstructure in suppressing interictal spikes in Electrical Status Epilepticus during Sleep. Clin. Neurophysiol..

[80] Campana C., Zubler F., Gibbs S., de Carli F., Proserpio P., Rubino A., Cossu M., Tassi L., Schindler K., Nobili L. (2017). Suppression of interictal spikes during phasic rapid eye movement sleep: A quantitative stereo-electroencephalography study. J. Sleep Res..

[81] Kang X., Boly M., Findlay G., Jones B., Gjini K., Maganti R., Struck A.F. (2020). Quantitative spatio-temporal characterization of epileptic spikes using high density EEG: Differences between NREM sleep and REM sleep. Sci. Rep..

[82] Rocamora R., Andrzejak R.G., Jiménez-Conde J., Elger C.E. (2013). Sleep modulation of epileptic activity in mesial and neocortical temporal lobe epilepsy: A study with depth and subdural electrodes. Epilepsy Behav..

[83] Zubler F., Rubino A., Russo G.L., Schindler K., Nobili L. (2017). Correlating Interictal Spikes with Sigma and Delta Dynamics during Non-Rapid-Eye-Movement-Sleep. Front. Neurol..

[84] Frauscher B., von Ellenrieder N., Ferrari-Marinho T., Avoli M., Dubeau F., Gotman J. (2015). Facilitation of epileptic activity during sleep is mediated by high amplitude slow waves. Brain.

[85] Malow B.A., Lin X., Kushwaha R., Aldrich M.S. (1998). Interictal Spiking Increases with Sleep Depth in Temporal Lobe Epilepsy. Epilepsia.

[86] Sheybani L., Mégevand P., Spinelli L., Bénar C.G., Momjian S., Seeck M., Quairiaux C., Kleinschmidt A., Vulliémoz S. (2021). Slow oscillations open susceptible time windows for epileptic discharges. Epilepsia.

[87] Sheybani L., Vivekananda U., Rodionov R., Diehl B., Chowdhury F.A., McEvoy A.W., Miserocchi A., Bisby J.A., Bush D., Burgess N. (2023). Wake slow waves in focal human epilepsy impact network activity and cognition. Nat. Commun..

[88] Grahnstedt S. (1986). Sleep deprivation and kindled seizures. Exp. Neurol..

